# The use of pyloric exclusion for treating duodenal trauma: case series

**DOI:** 10.1590/S1516-31802008000600009

**Published:** 2008-11-06

**Authors:** Gustavo Pereira Fraga, Guilherme Biazotto, José Benedito Bortoto, Nelson Adami Andreollo, Mario Mantovani

**Keywords:** Duodenum, Wounds and injuries, Sutures, Morbidity, Pancreatitis, Gastroenterostomy, Duodeno, Ferimentos e lesões, Suturas, Morbidade, Pancreatite, Gastroenterostomia

## Abstract

**CONTEXT AND OBJECTIVES:**

Significant controversy exists regarding the best surgical treatment for complex duodenal injuries. The aims of this study were to report on a series of eight cases of duodenal repairs using pyloric exclusion and to describe reported complications or improvements in clinical outcomes among patients with complex duodenal trauma.

**DESIGN AND SETTING:**

Cross-sectional study followed by a case series in a university hospital.

**METHODS:**

Data on eight patients with duodenal trauma who underwent pyloric exclusion over a 17.5 year period were collected and analyzed.

**RESULTS:**

The causes of the injuries included penetrating gunshot wounds (GSW) in five patients and motor vehicle accidents (blunt trauma) in three patients. The time elapsed until surgery was longer in the blunt trauma group, while in one patient, the gunshot injury was initially missed and thus the procedure was carried out 36 hours after the original injury. The injuries were grade III (50%) or IV (50%) and the morbidity rate was 87.5%. Four patients (50%) died during the postoperative period from complications, including hypovolemic shock (one patient), sepsis (peritonitis following the missed injury) and pancreatitis with an anastomotic fistula (two patients).

**CONCLUSIONS:**

Pyloric exclusion was associated with multiple complications and a high mortality rate. This surgical technique is indicated for rare cases of complex injury to the duodenum and the surgeon should be aware that treatment with a minimalistic approach, with only primary repair, may be ideal.

## RESUMO

**CONTEXTO E OBJETIVOS:**

Há controvérsias a respeito do melhor tratamento cirúrgico para as lesões duodenais complexas. O objetivo deste estudo é relatar uma série de oito casos de reparo duodenal utilizando a exclusão pilórica e descrever a evolução dos pacientes com trauma duodenal complexo submetidos a este procedimento.

**TIPO DE ESTUDO E LOCAL:**

Estudo transversal e descrição de série de casos em hospital universitário.

**MÉTODOS:**

Foram coletadas e analisadas as informações de oito pacientes com trauma duodenal submetidos à exclusão pilórica em um período de 17,5 anos.

**RESULTADOS:**

Os mecanismos de trauma envolvidos foram ferimentos por projétil de arma de fogo em cinco pacientes e acidente automobilístico em três pacientes. O atraso do tratamento cirúrgico foi maior nos pacientes vítimas de trauma fechado, e em um paciente a lesão pelo projétil passou despercebida, sendo o procedimento cirúrgico realizado em nova laparotomia após 36 horas. As lesões duodenais encontradas foram grau III (50%) ou grau IV (50%), e a taxa de morbidade foi de 87,5%. Quatro pacientes (50%) morreram durante o período pós-operatório, de complicações como choque hipovolêmico (um caso), sepse (peritonite por ferimento não identificado), ou pancreatite associada à fístula da anastomose (dois casos).

**CONCLUSÕES:**

A exclusão pilórica esteve associada à alta taxa de morbimortalidade. Esta técnica cirúrgica deve ser indicada em poucos casos de lesão complexa de duodeno e o cirurgião deve saber que, frente a uma lesão duodenal, a sutura primária pode ser o melhor tratamento.

## INTRODUCTION

Because of its retroperitoneal location, injuries to the duodenum are relatively uncommon, occurring in only 3 to 5% of all abdominal injuries.^1-3^ The majority of duodenal injuries are caused by penetrating trauma that requires immediate exploratory laparotomy. Blunt injuries are more difficult to treat, mainly because of the difficulty in making a timely diagnosis.^3,4^ The vast majority of duodenal injuries can be managed by simple repair.^1-4^ Repair of multiple or delayed injuries often presents a technical challenge, and a variety of techniques have been described. The use of duodenal diversion through gastrojejunostomy was originally conceived in the early 1900s,^5^ but the simplified technique of pyloric exclusion was devised by Jordan and first reported by Vaughan et al.^6^ in 1977. This procedure consists of primary repair of the duodenal wound, closure of the pylorus through gastrotomy and gastrojejunostomy at the site of the gastrostomy.

Pyloric exclusion has been recommended in selected patients with complicated duodenal injury because it decreases the morbidity associated with dehiscence and fistula formation. However, the current philosophy for the management of pancreaticoduodenal injuries is that less treatment is probably the best treatment.^7^

## OBJECTIVE

The purpose of this study was to report on a series of eight cases of duodenal repairs using pyloric exclusion and to describe reported complications or improvements in clinical outcomes among patients with complex duodenal trauma.

## METHODS

The study design was cross-sectional followed by a report on eight cases of duodenal repairs using pyloric exclusion. A total of 93 patients with penetrating or blunt abdominal trauma and duodenal injury were admitted to the Division of Trauma Surgery of Universidade Estadual de Campinas (Unicamp), a large teaching hospital in a metropolitan area of 2.4 million people, between January 1990 and June 2007. All of the known cases of duodenal trauma among these patients were reviewed. The data were entered into a protocol and analyzed using the Epi-Info 6.04 computer software. Institutional ethics committee approval was obtained. The data collected included demographics, gender, age, mechanism of injury, admission vital signs, time elapsed between injury and operation, site and grade of duodenal injury, associated organ injuries, surgical procedure used, presence of complications (including duodenal fistula) and mortality. The trauma indices used included the Revised Trauma Score (RTS),^8^ Injury Severity Score (ISS)^9^ and Abdominal Trauma Index (ATI),^10^ and duodenal injuries were classified using the American Association for the Surgery of Trauma - Organ Injury Scale (AAST-OIS).^11^

For the operation, a midline incision was performed. The first priority was to control life-threatening hemorrhage from vascular structures or parenchymatous organs, followed by controlling the sources of gastrointestinal spillage. The duodenum was explored and mobilized by means of a Kocher maneuver, a Cattell-Braasch maneuver, or both. The injuries were graded and surgical repair was dictated by the surgeon's judgment. The decision to use pyloric exclusion was based on the degree of duodenal injury, extent of multiple organ involvement, degree of edema and friability of the duodenum, time elapsed between injury and treatment and the general condition of the patient.

**Figure 1:**
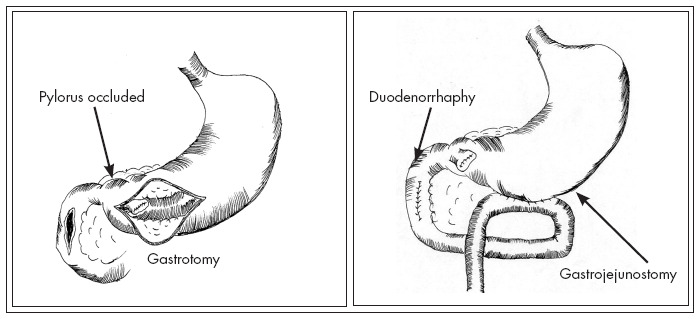
Pyloric exclusion procedure.^12^

Primary duodenal repair was attempted after debridement of the edges of the perforation in gunshot wounds (GSWs), and one-layer interrupted seromuscular duodenorrhaphy was used. The pylorus was occluded with nonabsorbable suture material through gastrotomy in a portion of the distal stomach, and side-to-side gastrojejunostomy was performed at the site of the gastrostomy (Figure 1).^12^ Vagotomy was not performed. A right upper quadrant drain was placed in all eight patients.

The minimum follow-up on the patients was six months.

## RESULTS

**Figure 2:**
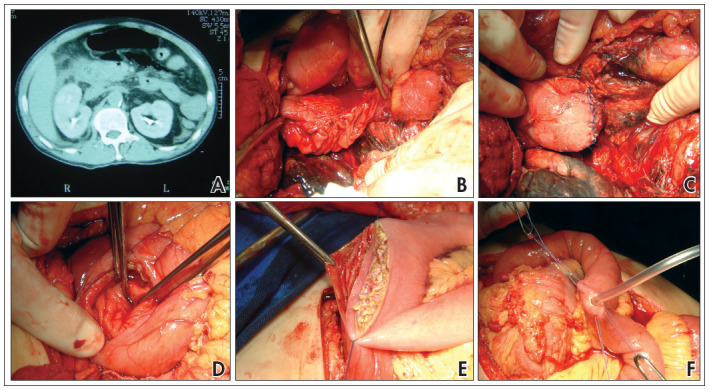
(A) Computed tomography showing air in retroperitoneum; (B) grade IV duodenal injury in third portion; (C) duodenal repair; (D) pylorus closed; (E) gastrojejunostomy; (F) feeding jejunostomy.

Over the 17½-year period, eight patients underwent pyloric exclusion (19% of the 42 patients with duodenal injury ≥ grade III according to AAST-OIS). Seven of these patients were male, and the patients’ mean age was 34.9 years (range: 18 to 55 years). The causes of injury included penetrating GSWs in five patients and blunt trauma from vehicular accidents in three patients. The mean systolic blood pressure on admission was 113 ± 18 mmHg and RTS was a maximum of 7.84 in seven patients and 2.77 in one. The time elapsed from admission to the surgical intervention ranged from 15 minutes to 18 hours and was longer in the blunt trauma group (Table 1). Computed tomography (CT) showed duodenal perforation in three blunt trauma patients (Figure 2). In one GSW patient with a large retroperitoneal hematoma, the posterior duodenal injury was missed in the first laparotomy and thus the procedure was carried out 36 hours post-injury. The most common injury site was the second portion of the duodenum, in five cases (62.5%) and none of these involved the ampullary complex. The remaining three patients had injuries in the third portion. Four patients had grade III injuries and four had grade IV. Associated abdominal injuries were identified in all of the patients and are listed in Table 1. The mean ISS was 20.1 ± 4.1 and the mean ATI was 30 ± 11.2.

**Table 1:** Summary of patients who underwent pyloric exclusion

Patient	Mechanism	RTS	Time elapsed between admission and surgery	Grade of injury	Associated abdominal injuries	ATI
1	Blunt	7.84	18 hours	IV	Pancreas	22
2	GSW	7.84	40 minutes*	III	Colon, liver, pancreas	47
3	GSW	7.84	40 minutes	III	Colon, liver, stomach	41
4	Blunt	7.84	3 hours	IV	Pancreas	21
5	GSW	7.84	20 minutes	III	Liver	20
6	GSW	7.84	50 minutes	IV	Kidney, liver	28
7	GSW	2.77	15 minutes	III	Colon, kidney, small bowel	41
8	Blunt	7.84	7 hours	IV	Pancreas	20

*RTS = revised trauma score; ATI = abdominal trauma index; GSW = gunshot wounds.*

* *pyloric exclusion performed at reoperation after 36 hours (missed injury).*

Feeding jejunostomy was performed in four patients (Figure 2). Six patients received a blood transfusion and the volume of packed red blood cells (PRBCs) was 1050 ± 1100 ml. Postoperative complications were common (morbidity of 87.5%; Table 2). Mortality was 50% (four patients) and, in three of these patients, it was related to pancreaticoduodenal injury. The overall mortality due to duodenal injuries out of the total pool of 93 patients was 28%.

**Table 2:** Postoperative complications and outcome

Patient	Complications	Reoperation	LOS	Survival
1	Pancreatic fistula	No	17 days	Yes
2	Missed duodenal injury, sepsis, MODS	Yes, repair and pyloric exclusion	3 days	No
3	Hepatic abscess, sepsis	No	30 days	Yes
4	Duodenal fistula, abdominal abscess	Yes, drainage of abscess	42 days	Yes
5	None	No	8 days	Yes
6	Pancreatitis, gastrojejunostomy fistula, sepsis, MODS	Yes, treatment of intra-abdominal infection	14 days	No
7	Hemorrhagic shock, coagulopathy	No	12 hours	No
8	ACS, pancreatitis, duodenal fistula, MODS	Yes, decompressive laparotomy	3 days	No

*LOS = length of stay; MODS = multiple organ dysfunction syndrome; ACS = abdominal compartment syndrome.*

## DISCUSSION

The management of duodenal injuries remains controversial, and this field lacks a consensus regarding what the optimal treatment should be. Approximately 70% to 85% of all duodenal injuries can be repaired safely by primary repair.^2,3,13,14^ Patients with severe duodenal injuries should be considered candidates for more complex duodenal repairs, such as duodenal diverticulization or pyloric exclusion. However, there is no clear definition regarding when these procedures should be indicated and which duodenal injuries should be considered severe. Certain factors may lead surgeons to consider an injury severe and order a complex procedure, including blunt trauma or bullet wounds, delay to repairs exceeding 24 hours, injury of the first or second portions of the duodenum, duodenal injuries of AAST-OIS grade ≥ III, associated injuries to the pancreas or common bile duct (or both) and compromised blood supply to the duodenum.^1,2,4,13,15^

The decision to use pyloric exclusion to repair a duodenal injury is multifactorial. This procedure appears to offer the best combination of limited surgery in cases of severely injured patients, with effective exclusion of the duodenum until after healing has occurred. Many authors have advocated the use of pyloric exclusion and have considered it to be the procedure of choice for patients with severe duodenal trauma.^6,7,16-20^ In our study, pyloric exclusion was performed on 8.6% of all the patients with duodenal injury, and we believe that the procedure was a reasonable management method, except in one patient with hypovolemic shock.

Vaughan et al.^6^ used pyloric exclusion on 75 patients selected from a total of 175 (42.9%) presenting with duodenal trauma, and had a mortality rate of 19% and a fistula formation rate of 5%. In another study by Martin et al.,^16^ including 313 patients with duodenal trauma, 128 (41%) with severe duodenal injuries were treated with pyloric exclusion and the duodenal fistula rate was 5.5%; two deaths due to fistulas occurred. Degiannis et al.^18^ studied pyloric exclusion for treating severe penetrating injuries of the duodenum, with a postoperative fistula rate of 43% (6/14) among patients who received only primary repair and 12% (2/17) among patients for whom pyloric exclusion was added to the surgical treatment. The authors concluded that grade III duodenal injuries due to gunshot wounds should always be treated with pyloric exclusion.^18^ Cogbill et al.^13^ reported 164 duodenal injuries, among which 27 patients (16.5%) were managed with pyloric exclusion. In their study, only two patients died secondary to duodenal complications (repair dehiscence and sepsis), thus suggesting that pyloric exclusion is a useful adjunct for more complex injuries.

Vagotomy is not usually part of this surgical procedure.^6,16,18^ Buck et al.^17^ described a study on 17 patients with severe pancreaticoduodenal injuries who underwent pyloric exclusion. Vagotomy was performed on eight of those patients. These authors reported high incidence of marginal ulcerations, and two patients without vagotomy presented with either perforations or bleeding and required reoperation. This complication rate was significantly higher than what had previously been reported, and the authors recommended that vagotomy should be added to pyloric exclusion at the time of the initial procedure.^17^ Ginzburg et al.^19^ performed pyloric exclusion without gastrojejunostomy on four patients, in order to avoid the extensive surgical repair required, so that they could focus on all the other associated injuries. They observed that spontaneous opening of the pylorus occurred and that the mean hospital stay was 29 days, thus concluding that gastrojejunostomy should not be used routinely on patients undergoing pyloric exclusion.^19^

In an experimental study on 30 rats subjected to pyloric exclusion with different occlusion suture materials and gastrojejunostomy with or without vagotomy, it was observed that nonabsorbable sutures maintained the pyloric closure for a longer duration (36.3 ± 11.6 days) and that vagotomy reduced the gastric inflammation without influencing the time when the pylorus should be reopened.^21^ In another study from our laboratory, Pierro et al.^12^ compared the results from primary repair versus repair associated with pyloric exclusion and gastrojejunostomy to treat complex duodenal injuries in 24 dogs. They did not observe any differences in the incidence of duodenal stenosis, fistula formation, intra-abdominal abscess or death between the two groups, but pyloric exclusion was a longer and more traumatic procedure, and it resulted in greater weight loss and increased incidence of vomiting among the animals.^12^

Primary repair, pyloric exclusion without vagotomy and gastrojejunostomy were used in all the patients in this study. Duodenal injuries have been associated with high morbidity, ranging from 38% to 100%, with an average of 63.7%.^2^ In the present study, only one patient (12.5%) was discharged without complications after eight days. The other three surviving patients suffered from abdominal infections (one hepatic abscess in a patient with associated colonic and gastric injury and one case of intra-abdominal abscess) or fistula development (one pancreatic and another duodenal). In the literature, duodenal fistula rates range from 0% to 16.2%, with an average incidence of 6.6%.^2,3,13,15^

In the current study, after the follow-up period of six months, the overall mortality rate for all duodenal injuries was 28% and 50% for the patients who underwent the pyloric exclusion procedure. We believe that this technique should not be used for patients with hypovolemic shock, for whom a faster procedure should be chosen. The patient with delayed surgical intervention because of an undetected injury developed peritonitis and ultimately died; this was directly attributable to the missed injury. Two other patients developed severe acute pancreatitis and anastomotic fistulas, related to duodenopancreatic trauma and sepsis. One 55-year-old patient treated recently (Figure 2) presented with a complex duodenal injury caused by blunt trauma and underwent surgery seven hours later. Twenty-four hours later, this patient presented multiple organ dysfunction syndrome in the abdominal compartment. During the decompressive laparotomy, we identified pancreatitis, and the patient developed a duodenal fistula and died on the third postoperative day. The overall mortality rate for duodenal injuries remains between 5.3% and 30%, but injuries to the duodenum itself are responsible for a mortality rate of about 10%.^1,2,4^

A recent study by Seamon et al.^7^ examined patients with penetrating duodenal injuries of grade ≥ II and pancreaticoduodenal injuries, excluding patients who died within 48 hours due to massive associated injuries. Fifteen out of their 29 patients were treated without pyloric exclusion and 14 with exclusion, and the groups were similar with regard to sex, age, mechanism, hemodynamic stability, injury grade (a trend toward greater injury severity was noted in the pyloric exclusion group), ISS, associated abdominal injuries and mortality rates. None of these patients suffered a duodenal fistula. These authors reported a trend towards a higher overall complication rate in the pyloric exclusion group (71% versus 33%), although this difference was not statistically significant. The same pattern was observed for the pancreatic fistula rate (40% versus 0%) and the length of hospital stay (24.3 days versus 13.5 days), and the in-hospital mortality rates were similar in the two populations (21% versus 7%). The authors concluded that simple repair without pyloric exclusion was both adequate and safe for most penetrating duodenal injuries.^7^ Their report presented significant study limitations, as recognized by the authors. It was retrospective and the patients were not randomized, thus suggesting that pyloric exclusion could have been applied to higher-risk patients. The same could have occurred in the present study. In a letter to the editor commenting on the review by Seamon et al.,^7^ Kashuk and Moore^22^ questioned their conclusion that pyloric exclusion should be abandoned, and pointed out that the review did not have sufficient scientific basis to justify abandoning this procedure.

## CONCLUSIONS

On the basis of our findings, although our study included a relatively small population, we believe that the general rule that "less is better" should be taken into consideration. Surgeons should be able to choose the best surgical procedure for managing patients with this challenging problem characterized by complex duodenal injuries. The role of pyloric exclusion requires further investigation in large, randomized and prospective trials, but we conclude that the indications for its use should be restricted during procedure selection and that patients usually seem to be safely treated with primary repair.
